# Novel Perceptions on Chemical Profile and Biopharmaceutical Properties of *Mentha spicata* Extracts: Adding Missing Pieces to the Scientific Puzzle

**DOI:** 10.3390/plants11020233

**Published:** 2022-01-17

**Authors:** Gokhan Zengin, Gunes Ak, Ramazan Ceylan, Sengul Uysal, Eulogio Llorent-Martínez, Simonetta Cristina Di Simone, Monica Rapino, Alessandra Acquaviva, Maria Loreta Libero, Annalisa Chiavaroli, Lucia Recinella, Sheila Leone, Luigi Brunetti, Amelia Cataldi, Giustino Orlando, Luigi Menghini, Claudio Ferrante, Marwa Balaha, Viviana di Giacomo

**Affiliations:** 1 Physiology and Biochemistry Research Laboratory, Department of Biology, Science Faculty, Selcuk University, 42130 Konya, Turkey; gokhanzengin@selcuk.edu.tr (G.Z.); akguneselcuk@gmail.com (G.A.); biyoram7@gmail.com (R.C.); 2 Halil Bayraktar Health Services Vocational College, Erciyes University, 38280 Kayseri, Turkey; senguluysal@erciyes.edu.tr; 3 Drug Application and Research Center, Erciyes University, 38280 Kayseri, Turkey; 4 Department of Physical and Analytical Chemistry, Campus Las Lagunillas, University of Jaén, E-23071 Jaen, Spain; ellorent@ujaen.es; 5 Botanic Garden “Giardino dei Semplici”, Department of Pharmacy, “Gabriele d’Annunzio” University, Via dei Vestini 31, 66100 Chieti, Italy; simonetta.disimone@unich.it (S.C.D.S.); alessandra.acquaviva@unich.it (A.A.); maria.libero@unich.it (M.L.L.); annalisa.chiavaroli@unich.it (A.C.); lucia.recinella@unich.it (L.R.); sheila.leone@unich.it (S.L.); luigi.brunetti@unich.it (L.B.); amelia.cataldi@unich.it (A.C.); giustino.orlando@unich.it (G.O.); luigi.menghini@unich.it (L.M.); marwa.balaha@unich.it (M.B.); viviana.digiacomo@unich.it (V.d.G.); 6 Genetic Molecular Institute of CNR, Unit of Chieti, “Gabriele d’Annunzio” University, Via dei Vestini 31, 66100 Chieti, Italy; m.rapino@unich.it; 7 Department of Pharmaceutical Chemistry, Faculty of Pharmacy, Kafrelsheikh University, Kafr El Sheikh 33516, Egypt

**Keywords:** *Mentha spicata*, antioxidants, sagerinic acid, enzyme inhibitor, neuromodulators

## Abstract

*Mentha spicata* is one of the most popular species in the genus, and it is of great interest as a gastrointestinal and sedative agent in the folk medicine system. In this study, different *M. spicata* extracts, obtained by the use of four solvents (hexane, chloroform, acetone and acetone/water) were chemically characterized using HPLC-ESI-MS ^n^, which allowed for identification of 27 phenolic compounds. The extracts’ antioxidant and enzyme inhibitory properties were investigated. In addition, neuroprotective effects were evaluated in hypothalamic HypoE22 cells, and the ability of the extracts to prevent the hydrogen peroxide-induced degradation of dopamine and serotonin was observed. The best antioxidant effect was achieved for all the extraction methods using acetone/water as a solvent. These extracts were the richest in acacetin, eriodictyol, hesperidin, sagerinic acid, naringenin, luteolin, chlorogenic acid, chrysoeriol and apigenin. The intrinsic antioxidant and enzyme inhibition properties of the acetone/water extract could also explain, albeit partially, its efficacy in preventing prostaglandin E_2_ overproduction and dopamine depletion (82.9% turnover reduction) in HypoE22 cells exposed to hydrogen peroxide. Thus, our observations can provide a scientific confirmation of the neuromodulatory and neuroprotective effects of *M. spicata*.

## 1. Introduction

*Mentha* is one of the most important genera of the Lamiaceae family, and its members are generally common in the temperate zone. The genus *Mentha* is represented worldwide with about 38 species [1] and in Turkey with 13 taxa [2]. The members of the genus *Mentha* contain important amounts of essential oils (EOs) [3,4], and people have long used them in tea, as aromatic agents and for therapeutic purposes [5]. *Mentha* species are used to treat gastrointestinal and respiratory diseases and are also employed as carminative, anti-spasmodic and sedative agents [1,6,7]. Indeed, in a recent article, Yousuf et al. [8] reported an antidepressant effect of the essential oil from *M. arvensis*. Similarly, the anesthetic potential of *M. piperita* was demonstrated by Brandão et al. [9]. Interestingly, several phytochemical studies showed that the members of the genus *Mentha* contain important classes of volatile and phenolic components [10,11,12,13,14,15,16,17]. In particular, the essential oils were reported to be cytotoxic and antibacterial agents, whereas polar extracts showed antioxidant properties [7,18,19,20,21,22,23]. Furthermore, the neuroprotective effects of phenolic compounds from *Mentha* species were demonstrated in preclinical experimental models of neuroinflammation and neurodegeneration [24,25].

*Mentha spicata* is one of the most common species in the genus *Mentha*, and several publications have acknowledged its biological and chemical potential [1]. *Mentha spicata* is a perennial herbaceous plant of great interest in the folk medicine system [1], especially for treating gastrointestinal disorders [7,26,27,28]. Other traditional ethnopharmacological uses include the treatment of respiratory disorders [29] and diabetes [30]. In this regard, fresh and dried leaves are used in different forms (i.e., decoctions, tinctures and tablets), whereas the whole plant and EOs are common ingredients in food, cosmetic and pharmaceutical products [1,3]. Like other *Mentha* species, *M. spicata* possesses diverse biological properties [1]; the EOs showed antibacterial, antimycotic, antilarvicidal and cytotoxic effects [31,32,33,34,35], while antidiabetic, anti-inflammatory, hepatoprotective and neuroprotective effects were exerted by different polarity extracts [36,37,38]. However, a potential neurotoxicity of *M. spicata* was described in the central nervous system (CNS), especially at the hypothalamic level [39]. Therefore, a deep investigation of the chemical composition and biological properties of *M. spicata* extracts, especially in terms of biocompatibility, could support the traditional uses and also lead to the development of innovative applications. 

Consequently, extracts obtained by using four different solvents (hexane, chloroform, acetone and acetone/water) and three extraction methods (homogenizer-assisted (HAE), ultrasound-assisted (UAE) and maceration (MAC)) were investigated in the present study. Hexane is a non-polar solvent which is able to extract non-polar compounds such as terpenes or terpenoids. Acetone is a non-toxic solvent that can be easily mixed with water in order to achieve the highest polarity. However, although there are several studies on *M. spicata* alcoholic and hydroalcoholic extracts [40,41,42], investigations on acetone and acetone/water extracts from *M. spicata* are limited. Therefore, the present paper aims to gain new knowledge about the choice of extraction solvents with *M. spicata.* The qualitative and quantitative chemical characterization of the tested extracts was carried out by using the HPLC-ESI-MS ^n^ technique. To determine the biological properties of the tested extracts, antioxidant and enzyme-inhibiting properties were evaluated in a cell-free model, whereas neuromodulatory and neuroprotective effects were investigated in hypothalamic HypoE22 cells. An in silico investigation was finally carried out with the aim to identify putative targets at the basis of the extracts’ bio-pharmacological effects. The results obtained could shed a powerful light on the path from natural resources to pharmaceutical products obtained from *M. spicata*.

## 2. Results and Discussion

### 2.1. Total Phenolic and Flavonoid Content

Recent studies have shown that extraction methods and solvents influence the presence and concentration of phenolics in the extracts [12,43,44,45]. Therefore, the extraction is a crucial step in phytochemical studies. In the present research, the effects of extraction methods and/or solvents on the total content of phenols and flavonoids in *M. spicata* extracts were first evaluated by colorimetric methods (Table 1). Acetone/water was found to be the best choice among the tested solvents regardless of the extraction method used, but the highest level in phenolic content was obtained from the combination with UAE (142.62 mg GAE/g extract). Among all extraction methods, the second highest values, ranging from 52.19 to 58.62 mg GAE/g extract, were recorded for the acetone extracts, while the lowest values were displayed in the hexane extracts obtained by MAC and UAE. The statistical evaluation showed significant differences in the total phenolic content in the extracts obtained from various extraction methods (*p* < 0.05).

Similar to total phenolic content, the highest content in flavonoids was found in the acetone/water extracts and the best value was detected in combination with HAE (92.20 mg RE/g) among the tested extraction methods. It was clearly established that total flavonoid levels showed significant differences due to both extraction solvents and methods (*p* < 0.05). We observed different results for each extraction method in terms of total flavonoid content. For HAE, acetone (14.43 mg RE/g) and chloroform (16.44 mg RE/g) extracts had a similar total flavonoid content, but no flavonoid could be detected in the hexane extract. Taken together, the total content of phenolics and flavonoids from *M. spicata* was significantly influenced by the method and solvent used in the extraction process. 

These findings are consistent with literature studies showing how polar extracts (methanol, ethanol or water) from *M. spicata* contain higher levels of phenolics compared to non-polar extracts [40,41,42]. In these studies, the quantification of phenolic compounds was mainly conducted through colorimetric assays. However, the validity of these methods in yielding accurate results is considered controversial [46]. For this reason, a punctual HPLC-ESI-MS ^n^ analysis was conducted to unravel the qualitative and quantitative composition of the phenolic components in the extracts taken into consideration.

### 2.2. HPLC-ESI-MS ^n^ Qualitative Analysis

The characterization of the phytochemicals (Table 2) was carried out by HPLC-ESI-MS ^n^ using the negative ion mode. Identification was performed using both analytical standards and bibliographic information. The compounds were numbered according to their elution order, keeping the same numbering in all extracts. A brief explanation of the characterization follows for those compounds not identified with analytical standards. Compound 1 was tentatively characterized as a disaccharide (probably diglucoside) due to the fragmentation pattern, typical of hexoside moieties [47]. Compound 3 presented [M-H]^−^ at *m*/*z* 315 and, after the loss of 162 Da (hexoside), yielded dihydroxybenzoic acid at *m*/*z* 153. Compounds 7 and 8 were eriodictyol glycosides. They suffered neutral losses of 308 Da (rutinoside) and 162 Da (hexoside) to yield the aglycone eriodictyol at *m*/*z* 287 (fragment ions at *m*/*z* 151 and 135) [48]. Compounds 11, 12 and 13 were luteolin glycosides. Compound 23 was identified as luteolin by comparison with an analytical standard, whereas the glycosides were characterized on the basis of the neutral losses of 162, 176 and 308 Da (hexoside, glucuronide and rutinoside, respectively). Compound 14 yielded naringenin at *m*/*z* 271 [48] after the loss of 308 Da, so it was characterized as naringenin-*O*-rutinoside. Compounds 15 and 18 were apigenin glycosides, showing the aglycone apigenin at *m/z* 269 (main fragment ion at *m*/*z* 225). Compound 17 showed [M-H]^−^ at *m*/*z* 717 and fragment ions at *m*/*z* 537 and 519. This fragmentation pattern was already reported for salvianolic acids B, E and L [49], but the exact isomer could not be identified. Compound 19 was characterized as chrysoeriol-*O*-rutinoside, previously reported in *M. spicata* [50]. Compound 24 was characterized as the aglycone chrysoeriol, while compound 20 was identified as sagerinic acid based on previous data on *M. spicata* [50]. Compound 21, with [M-H]^−^ at *m*/*z* 537, was tentatively characterized as salvianolic acid I based on bibliographic data [51]. Compound 22 suffered the loss of 308 Da to yield the aglycone acacetin at *m/z* 283 (main fragment ion at *m*/*z* 268) [48], so it was characterized as acacetin-*O*-rutinoside. Compound 25, with [M-H]^–^ at *m*/*z* 551 and fragment ions at *m*/*z* 519 and 359, was tentatively characterized as monomethyl lithospermate [52]. Compounds 26 and 27 were characterized as the oxylipins oxo-dihydroxy-octadecenoic acid and trihydroxy-octadecenoic acid on the basis of bibliographic information [53].

### 2.3. Distribution of Compounds between the Tested Extracts

**Figure 1:**
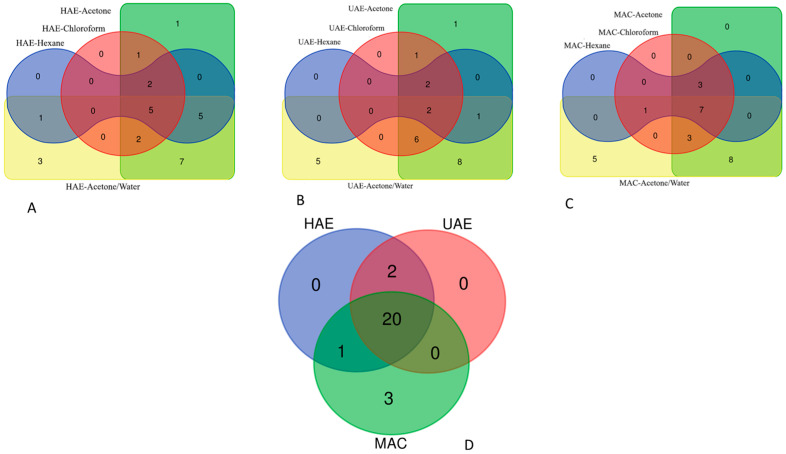
Venn diagram showing the number of common compounds found in the tested extracts. (**A**): Extracts obtained by homogenizer assisted extraction; (**B**): Extracts obtained by ultrasound assisted extraction; (**C**): Extracts obtained by maceration technique; (**D**): Comparison of acetone/water extracts obtained by the three different extraction methods.

To detect any difference in the presence of the identified compounds in the tested extracts, we used Venn diagrams (Figure 1). In addition, the comparison results between the extracts are given in Supplementary Materials (Tables S1–S4). The chemical composition of the tested extracts depends on the extraction solvents and methods. As expected, acetone and acetone/water extracts were the richest in terms of the variety of compounds present in them, regardless of the extraction method used. For example, 23 compounds were identified in acetone and acetone/water extracts obtained by HAE. On the other hand, the lowest number of compounds (5) was detected in UAE–hexane extract. These results indicate that *M. spicata* is rich in polar compounds. In all acetone/water extracts, 20 compounds were identified as common. Citric and caffeic acids were only detected in MAC–acetone/water extract. In acetone/water extracts, chrysoeriol and luteolin were only found in HAE and MAC (see Supplemental Materials).

### 2.4. Quantification of Phytochemicals by HPLC-DAD

The main components of the extracts were quantified by HPLC with UV detection. Commercial standards of caffeic acid (for sagerinic acid; 320 nm), chlorogenic acid (320 nm), hesperidin (for flavanones; 280 nm) and apigenin (for flavones; 350 nm) were used. Calibration graphs were constructed in the 0.5–100 mg/L range and Relative Standard Deviations lower than 5% were observed in all cases. The results are shown in Table 3. First, only the compounds found in acetone and acetone/water extracts could be quantified. The use of hexane and chloroform solvents provided a very low extraction yield, so the compounds’ concentrations were below the detection limit of the method. Second, to the best of our knowledge, although the main compounds have been previously reported in *M. spicata* or other *Mentha* species, this is the first quantitative report of the individual composition of phytochemicals in *M. spicata*. The most abundant compounds were flavonoids, mainly in their glycosilated form, although high concentrations of sagerinic acid (a rosmarinic acid derivative) were also found. For all extraction techniques, the use of acetone/water as an extractant was clearly more efficient than the use of water (see TIPC in Table 3). However, among the different extraction techniques, the overall extraction yields were similar. The contribution of flavonoids to the total phytochemicals ranged between 81 and 92%; the most abundant flavonoids were eriodictyol-*O*-rutinoside (compound 7) and luteolin-*O*-hexoside and luteolin-*O*-glucuronide (compounds 12 and 13). However, in the acetone/water extracts, the concentrations of sagerinic acid (compound 20) were similar to those of luteolin glycosides and approximately 50% of eriodictyol-*O*-rutinoside.

### 2.5. Antioxidant Properties

In recent studies, clinical and epidemiological data showed that oxidative stress is very closely linked to chronic metabolic and neurodegenerative diseases, and plant extracts have been studied for their potential role in contrasting the burden of oxidative stress occurring in these pathologies [54,55]. In the search for new protective agents, the antioxidant properties of *M. spicata* extracts were investigated by cell-free chemical methods (Table 4). The ability of the plant extracts to quench the two radicals ABTS and DPPH was evaluated as a measure of their antiradical potential. Acetone/water extracts showed significantly higher radical scavenging values with respect to all the other solvents used, while the lowest radical scavenging activity was found in hexane and chloroform extracts for both assays. In addition, the extraction method influences the antiradical potential of the extracts: the hexane extract has better results when combined with the HAE method, while when the solvent used is acetone/water, the radical scavenging value is significantly lower if the method used is maceration. Interestingly, these findings are in line with the respective total content in all phenols (Table 1).

The reducing properties of a plant extract reflect the ability to donate electrons whose transfer could result in a colour change, which can be detected by spectrophotometric methods such as CUPRAC and in FRAP. As can be seen in Table 4, the best reduction capacities in both assays were found in acetone/water extracts (456.73–782.24 mg TE/g in CUPRAC; 302.10–492.27 mg TE/g in FRAP), while the extraction with hexane always registers the lowest values in both tests. However, the reducing capacity of hexane extract is significantly higher in combination with HAE than with the other two methods. On the other hand, the UAE method results in the highest reducing power when the solvent is acetone/water. 

The phosphomolybdenum (PBD) assay is based on the ability of anti-oxidant compounds to reduce Mo (VI) to Mo (V). Since all antioxidants (phenolics and non-phenolics) play a reducing role in the PBD assay, this test could be considered as one of the assays describing the total antioxidant capacity of the extracts. In contrast to free radical scavenger and reducing power assays, each solvent registers different results when combined with a different extraction method. For example, among the extracts obtained by the homogenizer-assisted method, the best ability in the PBD assay was found in hexane extract (3.23 mmol TE/g). The two highest effects for ultrasound assisted extracts and maceration were determined by acetone/water (3.48 mmol TE/g) and acetone (2.54 mmol TE/g), respectively. In addition, all extracts from HAE and UAE showed statistical differences in the phosphomolybdenum assay (*p* < 0.05), while in MAC, no significant difference was observed between the chloroform (2.38 mmol TE/g) and acetone (2.54 mmol TE/g) extracts.

In the metal chelating assay, the ability of the extract to chelate transition metals, which is due to the inhibition of the production of hydroxyl radicals in the Fenton reaction, was measured. The hexane and acetone extracts by the homogenizer-assisted method showed the strongest metal chelating abilities, with values of 24.09 and 21.80 mg EDTAE/g, respectively. The acetone extract values were significantly higher than the other solvent when combined with both UAE and MAC methods (21.54 mg EDTAE/g and 20.18 mg EDTAE/g, respectively). In HAE, the lowest values were provided by the chloroform (3.30 mg EDTAE/g) and acetone/water (7.98 mg EDTAE/g) extracts, and they exhibited similar abilities (*p* > 0.05).

**Figure 2:**
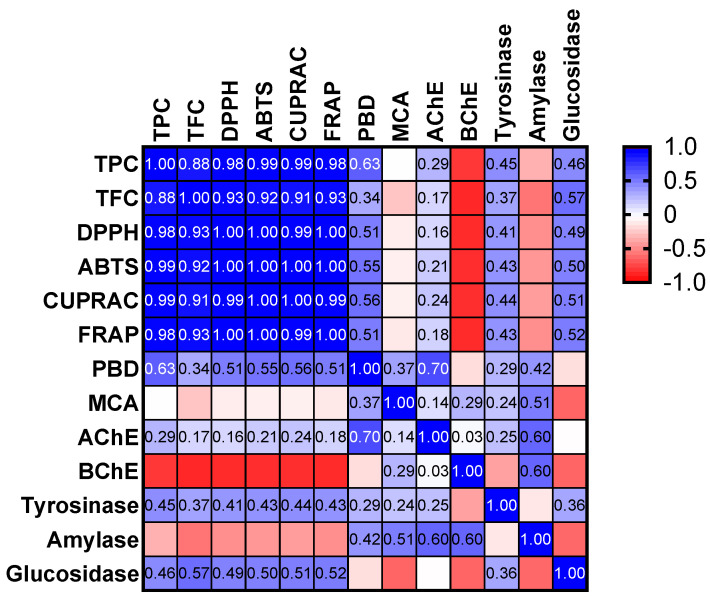
Pearson’s correlation values (R) in the biological activity assays performed; AChE, acetylcholinesterase; BChE, butyrylcholinesterase; CUPRAC, cupric ion reducing antioxidant capacity; DPPH, 1,1-diphenyl-2-picrylhydrazyl; FRAP, ferric ion reducing antioxidant power; MCA, metal chelating activity; PBD, phosphomolybdenum assay; TFC, total flavonoid content; TPC, total phenolic content.

When the results of the antioxidant assays are evaluated together, the free radical scavenging and the reducing power appear in line with their total phenolic content. This fact was also confirmed by Pearson’s correlation analysis, and the results are shown in Figure 2. High correlation values (R > 0.8) were observed between the total phenolics’ content and the results in most of the aforementioned assays. In agreement with our findings, several researchers reported strong correlation values between these assays in plant extracts [27,56,57]. However, we observed a low correlation value for PBD assay, and this fact could be explained by the presence in the extracts of other reducing molecules (peptides, sugars, etc.). Similarly, the low correlation value observed between the total phenolics and metal chelating ability might be explained by the action of the non-phenolic chelators in the tested extracts. Despite there being several studies about the antioxidant properties of *M. spicata* in the literature, little is known about the influence of the extraction method and the solvent (especially acetone/water) on the extract composition [58,59,60]. 

As for the quantitative analysis of chemical constituents in the tested extracts, acetone and acetone/water extracts contained more compounds than hexane and chloroform ones. For example, rutin and chlorogenic acid were only present in acetone/water extracts in the tested extraction methods. As shown in Table 3, the acetone/water extracts also contained higher concentrations of eriodictyol, luteolin and sagerinic acid. The observed antioxidant properties could be explained by the structure, the position and numbers of the hydroxyl groups and the glycosylation of the flavonoid ring. In particular, polyhydroxyl groups in the B ring of flavonoids could improve their radical scavenging abilities [61]. In addition to the organization of the B ring, the presence of a 2,3 double bond in the C ring and 3- or 5-hydroxyl groups could increase the antioxidative and, especially, radical scavenging potentials of the flavonoids [62]. Furthermore, the number of hydroxyl groups in phenolic acids could be related to their radical scavenging abilities. Sagerinic acid is derived from rosmarinic acid and the cyclic compound contains more hydroxyl groups than rosmarinic acid [63]. This fact can also improve its radical scavenging properties. In addition, the position and number of hydroxyl groups in flavonoid moieties could affect their metal chelating abilities. This fact was also reported by Mira et al. [64], who demonstrated the interaction of flavonoids with transition metals. 

### 2.6. Enzyme Inhibitory Properties

In the present study, the inhibitory properties of *M. spicata* extracts towards enzymes, known for playing master roles in diverse chronic and degenerative pathologies, were evaluated. Specifically, we measured the inhibition of the activity of α-amylase and α-glucosidase as targets of antidiabetic therapy, tyrosinase and cholinesterases (AChE and BChE), which play master roles in skin disorders and Alzheimer’s disease (AD), respectively [65,66,67,68,69,70]. The results are summarized in Table 5. Regarding the AChE inhibition, the best ability was observed in the chloroform extract obtained via ultrasound-assisted extraction, with an inhibitory effect corresponding to 4.84 mg GALAE/g extract. Generally, the chloroform extracts were more active than the extracts with other solvents, whereas the hexane extracts (from ultrasound-assisted extraction and maceration) were not active on AChE. Regarding the BChE inhibition, different results were obtained for each extraction technique. For example, the ability of the homogenizer-assisted extracts was as follows: hexane (8.70 mg GALAE/g) > chloroform (6.50 mg GALAE/g) > acetone (5.61 mg GALAE/g) > acetone/water (2.12 mg GALAE/g). Only the acetone/water extract obtained by maceration was not active on BChE activity. Tyrosinase is a key enzyme in melanin production and its inhibition is considered a key point in the management of hyperpigmentation problems. The best tyrosinase inhibitory effect (108.38 mg KAE/g) was shown by the acetone/water extract obtained by maceration. Acetone/water and acetone extracts were generally more active on tyrosinase than other solvent extracts, with the exception of homogenizer-assisted extracts. The ability to inhibit α-amylase and α-glucosidase was partially influenced by both the extraction technique and the solvent. The highest α-amylase inhibitory properties (0.86 mmol ACAE/g) were displayed by the acetone extract obtained via homogenizer-assisted extraction. In general, the lowest α-amylase inhibitory effects were shown by acetone/water extracts. Regarding the α-glucosidase inhibition, the inhibitory effect reached a maximum value of 1.66 mg ACAE/g and 1.62 mg GALAE/g for the acetone/water extracts obtained via homogenizer-assisted extraction and maceration, respectively. With the exception of ultrasound-assisted extraction, the lowest abilities were found in hexane extracts. 

### 2.7. In Vitro Neuromodulatory and Neuroprotective Effects of Mentha spicata Extracts

When the results of enzyme inhibition assays were correlated to the total phenolic content of the extracts, only α-glucosidase inhibitory effect was moderately correlated with the flavonoid content (R > 0.5). The correlation value can be associated with the presence of some flavonoids such as eriodictyol, luteolin and apigenin, which had higher concentrations in the acetone/water extracts. These compounds were described by several researchers as potent inhibitors of α-glucosidase [71,72]. Sohretoglu and Sari [72] investigated the glucosidase inhibitory properties of flavonoids and explained the structure–ability relationship very well. For example, glycosylation of the flavone ring could decrease glucosidase inhibitory effects. On the other hand, 3,4 dihydroxyl groups in the B ring of flavonoid moiety could increase the ability, as well as the 3-hydroxyl group in the C ring. At this point, the identified flavonoids and their derivatives could explain the observed glucosidase inhibitory properties. Indeed, the observed high glucosidase inhibitory properties of acetone/water extracts could be explained with the presence of flavonoids.

In addition to flavonoids, the phenolic acids, including caffeic, chlorogenic and sagerinic acids, could form the basis of the observed enzyme inhibitory properties. Several researchers reported that chlorogenic acid possesses a large spectrum of enzyme inhibitory properties [73,74,75,76]. For example, caffeic and chlorogenic acids were examined for cholinesterase inhibition properties, and the former showed a stronger ability than chlorogenic acid [73]. The authors reported that the number and position of the hydroxyl groups may be important in the interaction with the active sites of AChE and BChE. The quinic acid moiety of chlorogenic acid reduced its inhibition ability [73]. In addition, the hydroxyl group in the B ring of flavonoids can interact with the active centers of AChE and BChE, and thus the presence of these groups could be linked to their inhibitory properties [77]. In terms of tyrosinase inhibitory properties, flavonoids can be responsible for the observed abilities of the tested extracts. In a recent paper published by Obaid et al. [78], flavonoids have been reviewed as natural tyrosinase inhibitors. In the paper, some structural properties of flavonoids are linked to their tyrosinase inhibitory properties. For example, the hydroxyl groups of B ring could be attributed to the inhibitory properties, and keto-moiety in the C ring could also lead to the ability to chelate copper in the active site of tyrosinase. Interestingly, eriodictyol and luteolin have a 3-hydroxyl group in the B ring [78], and the presence of these compounds in the tested extracts could explain the observed anti-tyrosinase abilities. 

In the literature, several studies have examined the effects of the enzyme inhibitory properties of the members of the genus *Mentha*, including *M. spicata* [79,80,81,82]. However, the effects of extraction methods and solvents on enzyme inhibitory properties of *M. spicata* are still unexplored.

Considering the aforementioned biological properties of the extracts, *M. spicata* is considered a powerful source of natural antioxidants and enzyme inhibition agents, and the obtained results may open new avenues for future pharmaceutical applications.

**Figure 3:**
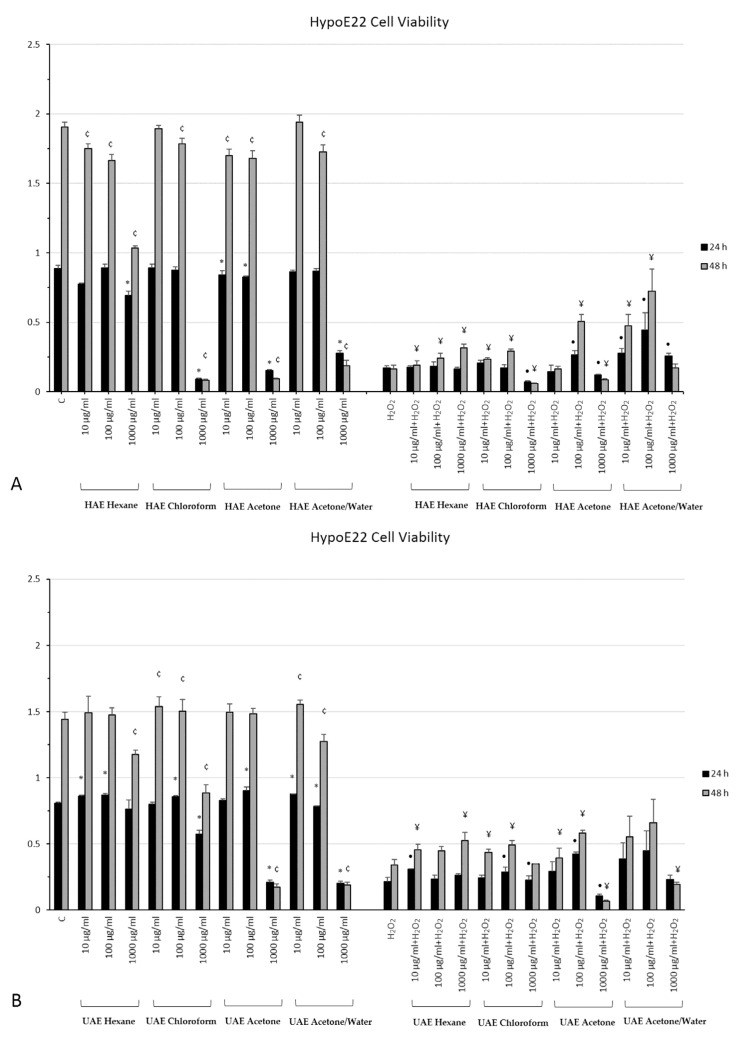

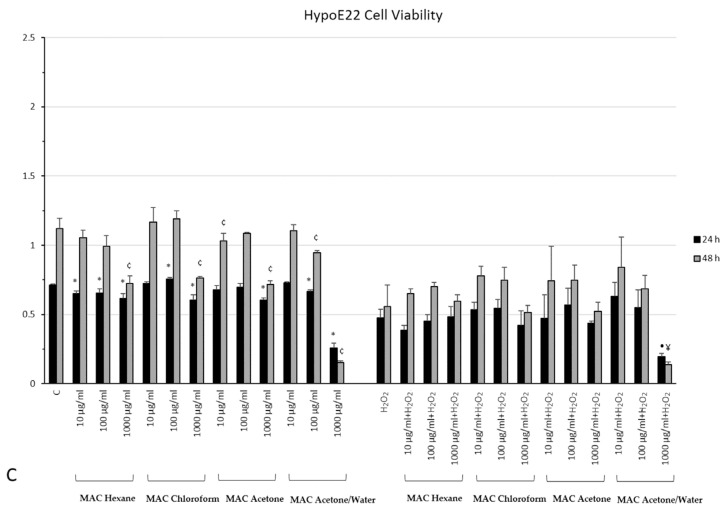
MTT assay of HypoE22 hypothalamic cell line exposed different concentrations (10–1000 μg/mL) of different *M. spicata* extracts for 24 and 48 h. Extracts with HAE (**A**), UAE (**B**) and MAC (**C**) methods are shown. The data graph bars are the mean ± SD (*n* = 3). * *p* < 0.05 vs. 24 h C; • *p* < 0.05 vs. 24 h H_2_O_2_; ¢ *p* < 0.05 vs. 48 h C; ¥ *p* < 0.05 vs. 48 h H_2_O_2_.

All twelve extracts were administered to HypoE22 cells at 10, 100 and 1000 µg/mL, and cell viability was evaluated both in basal condition and when cells were exposed to an oxidative stress, represented by 3 h exposure to H_2_O_2_ 300 µM (Figure 3A–C). Half of the extracts (HAE chloroform, HAE acetone, HAE acetone/water, UAE acetone, UAE acetone/water and MAC acetone/water) are highly cytotoxic at the highest concentration as well as in basal conditions, both at 24 and 48 h. The other six (HAE hexane, UAE hexane, UAE chloroform, MAC hexane, MAC chloroform and MAC acetone) are toxic at least after 48 h of treatment and at the highest concentration of 1000 µg/mL. Consequently, the highest concentration was not considered for further studies. No relevant effects were observed for the other concentrations of extracts administered when cells were cultivated in cell medium. As for the cells exposed to the oxidative stress and *M. spicata* extracts, almost all the plant extracts increased cell viability when affected by the oxidative stress, especially at the lowest concentrations (10 and 100 µg/mL). The HAE acetone/water extract, in particular, showed the most relevant effects in protecting the cells from death induced by H_2_O_2_. This extract was then chosen for further analysis.

**Figure 4:**
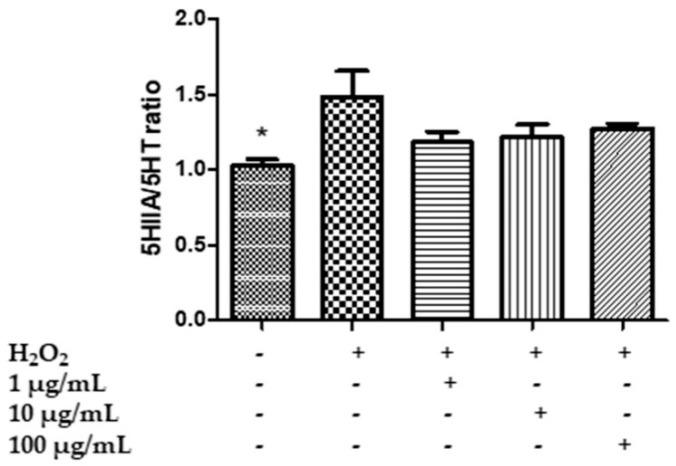
Effect of the HAE acetone water extract (1–100 µg/mL) on serotonin turnover, measured as 5HIIA/5-HT ratio, in hypothalamic HypoE22 cells exposed to H_2_O_2_ (µM). ANOVA *p* < 0.01; * *p* < 0.05 vs. H_2_O_2_.

**Figure 5:**
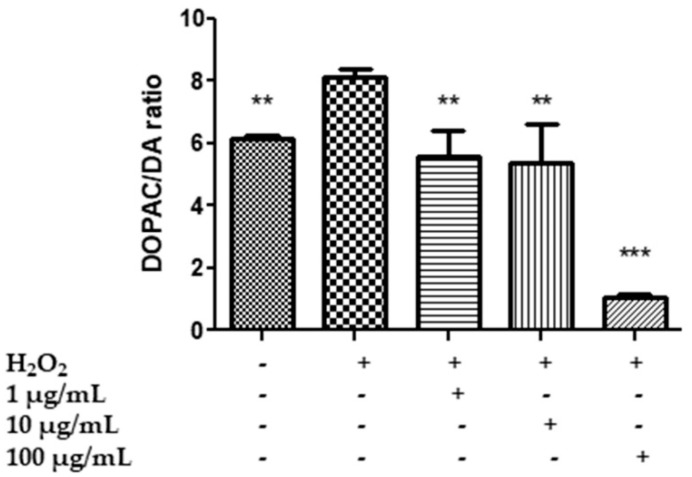
Effect of the HAE acetone water extract (1–100 µg/mL) on dopamine turnover, measured as DOPAC/DA ratio, in hypothalamic HypoE22 cells exposed to H_2_O_2_ (µM). ANOVA *p* < 0.0001; ** *p* < 0.01, *** *p* < 0.001 vs. H_2_O_2_.

**Figure 6:**
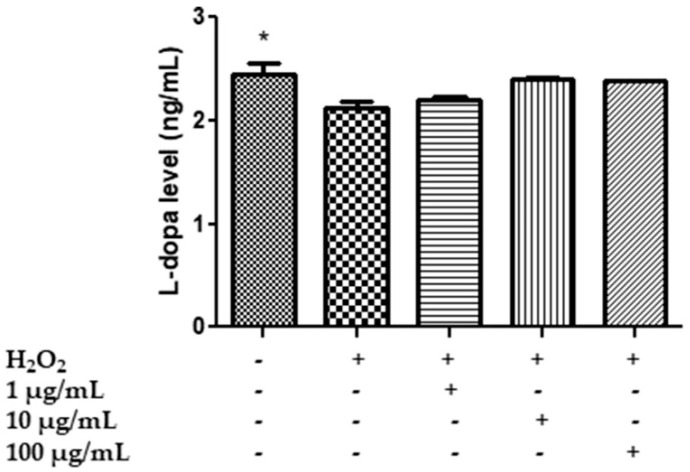
Effect of the HAE acetone water extract (1–100 µg/mL) on L-dopa level (ng/mL), in hypothalamic HypoE22 cells exposed to H_2_O_2_ (µM). ANOVA *p* < 0.01; * *p* < 0.05 vs. H_2_O_2_.

Specifically, HypoE22 cells were exposed to H_2_O_2_ 300 µM and treated with HAE acetone/water extract at 1–100 µg/mL for 48 h, and the extracellular levels of 5-HT, 5HIIA, DA, DOPAC and L-dopa were evaluated in the supernatants. The hydrogen peroxide stimulus was able to increase the turnover of 5-HT and DA, represented by the ratios of 5HIIA/5-HT (Figure 4) and the DOPAC/DA (Figure 5), and to reduce the levels of L-dopa (Figure 6), while the extract was effective, albeit partially, in preventing the neurotransmitter’s degradation. In the case of serotonin, the extract showed a trend in contrasting the hydrogen peroxide-induced increase in 5HIIA/5-HT, which is a reliable index of MAO-A activity [83]. However, the effect was not statistically significant. Similarly, the extract did not modify the level of L-dopa, an index of the activity of tyrosine hydroxylase, the rate limiting enzyme in DA biosynthesis [84]. On the other hand, the extract was able to prevent the increase in the DOPAC/DA ratio, which is a measure of MAO-B activity [83]. The inhibition of DA turnover by *M. spicata* extract suggested protective effects on DA signaling. These results are in line with previous investigations conducted on *Mentha piperita* and point to its efficacy in reducing 5-HT and DA turnover and anxiety in vivo [25]. However, neurotoxicity effects of *M. spicata* extracts were observed in different neural cells, including neuroblastoma and hypothalamic neurons [39,85]. In the case of hypothalamic cells, the toxicity of *M. spicata* extracts was related to the downregulation of antioxidant defence enzymes. However, this is in contrast, albeit partly, with the intrinsic scavenging/reducing, but also neuroprotective, effects showed by the present and previous studies [85]. This apparent discrepancy could be explained by the differences in the extraction methods and solvent as well as in the concentrations used. 

**Figure 7:**
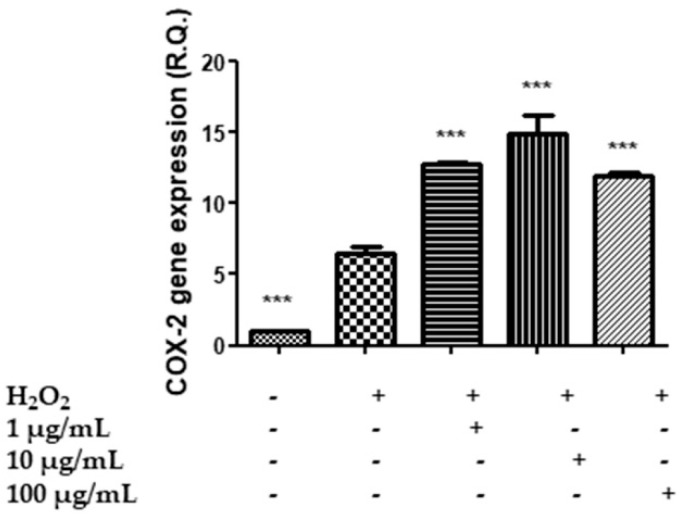
Effect of the HAE acetone water extract (1–100 µg/mL) on COX-2 gene expression in hypothalamic HypoE22 cells exposed to H_2_O_2_ (µM). ANOVA *p* < 0.0001; *** *p* < 0.001 vs. H_2_O_2_.

**Figure 8:**
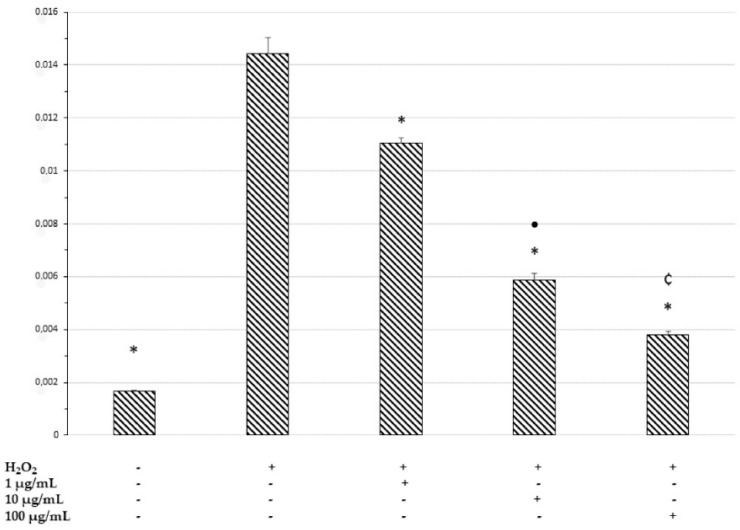
ELISA assay for PGE_2_ secretion of cultures at 48 h. The secretion levels are reported as pg/mL. The data shown are the mean (±SD) (*n* = 3). * *p* < 0.5 vs H_2_O_2,_ • *p* < 0.05 vs. 1 µg/mL + H_2_O_2_; ¢ *p* < 0.05 vs. 10 µg/mL + H_2_O_2_.

Therefore, in order to shed light on the role of *M. spicata* in the hypothalamus, the COX-2 gene expression and the levels of PGE_2_ were measured as markers of neuroinflammation. The COX-2 gene expression was increased by extract treatment (Figure 7). On the other hand, the release of PGE_2_, the main COX-2-deriving prostanoid, in the cell medium increases when the cells are exposed to H_2_O_2_, but the *M. spicata* extract exerts a concentration-dependent inhibition on the hydrogen peroxide-induced prostaglandin production (Figure 8). The inhibition of the PGE_2_ level suggests an anti-neuroinflammatory effect induced by the extract which is consistent, albeit partially, with the extract’s antiradical properties and the phenolic content. The inhibition of PGE_2_ level also agrees with the anti-inflammatory effects of *M. spicata* and *M. piperita* [86]. Moreover, it is sensible to highlight how the HAE acetone/water extract is the richest in terms of phenolics and the most active as a scavenging/reducing agent among all *M. spicata* extracts tested. Additionally, the opposite effects on PGE_2_ level and COX-2 gene expression suggest that the anti-inflammatory effect of the extract in the hypothalamus could derive, albeit partially, from the inhibition of the enzyme activity, rather than from a modulatory effect on the enzyme’s gene expression. 

### 2.8. In Silico Study

**Figure 9:**
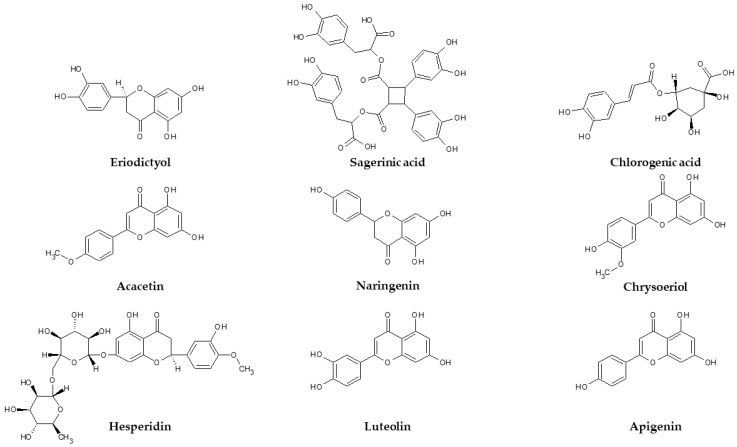
Structure of eriodictyol, acacetin, hesperidin, sagerinic acid, naringenin, luteolin, chlorogenic acid, chrysoeriol and apigenin.

**Figure 10:**
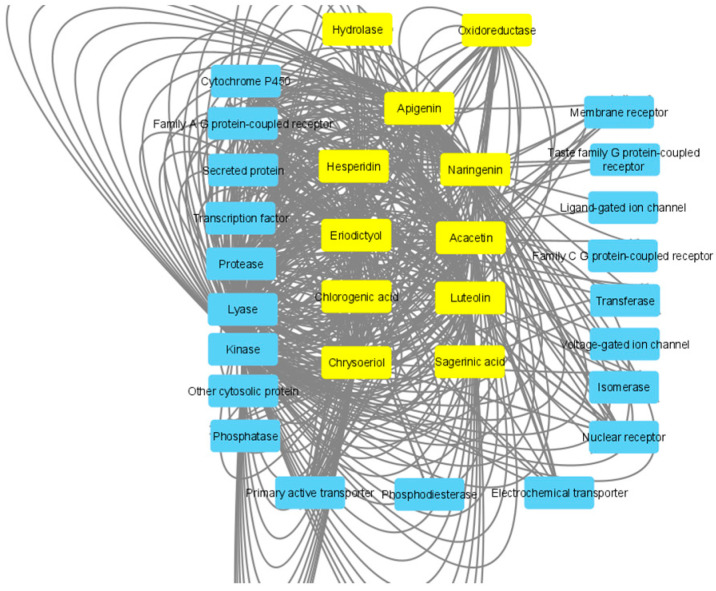
Pharmacological profile of phytocompounds identified through chromatographic analysis in the HAE acetone/water extract of *M. spicata*. Molecular targets were predicted through the SwissTargetPrediction platform and a components-targets analysis was carried out through Cytoscape software (3.7.2 version) on acacetin, eriodictyol, hesperidin, sagerinic acid, naringenin, luteolin, chlorogenic acid, chrysoeriol and apigenin.

Aiming to shed light on the mechanisms underlying the observed effects, an in silico prediction was conducted considering the prominent extract’s phytocompounds characterized by a well-defined structure, namely acacetin, hesperidin, eriodictyol, sagerinic acid, naringenin, luteolin, chlorogenic acid, chrysoeriol and apigenin (Figure 9). These compounds were predicted to interact with a wide number of protein families, including oxydoreductases and hydrolases (Figure 10). Additionally, in this context, the putative interactions with MAO-A, MAO-B, COX-2, tyrosinase and cholinesterases are consistent both with our present findings of enzyme inhibition exerted by the extract and literature data demonstrating the enzyme inhibition properties of the aforementioned phytochemicals [87,88,89,90,91,92,93].

## 3. Materials and Methods

### 3.1. Plant Materials

*Mentha spicata* subsp. *spicata* samples were collected in the city of Erzurum (Karabıyık village, Turkey), in the 2020 summer season (August). The plants were confirmed by a botanist in Selcuk University, Science Faculty, Konya, Turkey, and one voucher specimen (N. GA-20-001) has been deposited in the Department of Biology, Selcuk University. The aerial parts of the plant samples were dried in shade conditions for 10 days at room temperature. The plant samples were powdered by using a laboratory mill and the powdered plant samples were stored in dark conditions at room temperature. 

In the present work, we used hexane, chloroform, acetone and acetone/water (70%) as solvents. Homogenizer-assisted extraction (HAE), ultrasound-assisted extraction (UAE) and maceration (MAC) were used as extraction methods. Briefly, plant materials (10 g) were homogenized with 200 mL of solvents by using Ultra-turrax at 6000 g for 5 min in HAE. In UAE, the plant materials (10 g) were sonicated with 200 mL of solvents at 30 °C for 20 min. In MAC, the plant materials (10 g) were macerated with 200 mL of solvents at room temperature for 24 h. Then, all extracts were filtered and the solvents were removed using a rotary evaporator. All extracts were stored at 4 °C until analysis.

### 3.2. Total Phenolic and Flavonoid Content

Total phenolic content (TPC) and total flavonoid content (TFC) were determined according to previously described methods [94,95]. TPC was expressed as mg gallic acid equivalents (GAE)/g dry extract, whereas TFC was expressed as mg rutin equivalents (RE)/g dry extract. 

### 3.3. Chromatographic Analysis of the Extracts

Chromatographic analyses were performed with an Agilent Series 1100 HPLC system with a G1315B diode array detector (Agilent Technologies, Santa Clara, CA, USA) and an ion trap mass spectrometer (Esquire 6000, Bruker Daltonics, Madrid, Spain) with an electrospray interface operating in negative ion mode. Separation was performed in a Luna Omega Polar C_18_ analytical column (150 × 3.0 mm; 5 µm particle size, Phenomenex, Madrid, Spain) with a Polar C_18_ Security Guard cartridge (4 × 3.0 mm), both purchased from Phenomenex. Detailed chromatographic conditions are available in [96].

### 3.4. Antioxidant and Enzyme Inhibitory Assays

In the current work, the antioxidant effects of the tested extracts were detected by different assays [94]. The assays were 1,1-diphenyl-2-picrylhydrazyl (DPPH) and 2,2′-azino-bis(3-ethylbenzothiazoline) 6-sulfonic acid (ABTS) radical scavenging, cupric ion reducing antioxidant capacity (CUPRAC), ferric ion reducing antioxidant power (FRAP), metal chelating ability (MCA) and phosphomolybdenum assay (PDA). For the DPPH, ABTS, CUPRAC and FRAP assays, data were expressed as mg Trolox equivalents (TE)/g extract, whereas in MCA and PDA, mg EDTA equivalents (EDTAE)/g extract and mmol TE/g extract, respectively, were used. The experimental parts for acetylcholinesterase, butyrylcholinesterase, tyrosinase, α-amylase and α-glucosidase assays were previously provided. Galantamine was used as a positive control in cholinesterase assays and data were evaluated as mg galantamine equivalents (GALAE)/g extract. Kojic acid was used as a standard inhibitor in tyrosinase inhibitory assay and the results were expressed as mg kojic acid equivalents (KAE)/g extract [94,95]. Acarbose was selected as an inhibitor for α-amylase and α-glucosidase inhibitory assays and the results are given as mmol acarbose equivalents (ACAE)/g extract. The assays were performed in triplicate and the differences in the extracts were evaluated by ANOVA assays (Tukey’s test). The correlation analysis between TPC, TFC and biological activities was reported as a Pearson’s coefficient, calculated using GraphPad version 9.

### 3.5. Cell Culture and Treatment

The HypoE22 rat-hypothalamus cell line was purchased from Cedarlane Corporation (Burlington, ON, Canada) and cultured in Dulbecco’s Modified Eagle Medium (DMEM) supplemented with 10% (*v*/*v*) heat-inactivated fetal bovine serum and penicillin–streptomycin (100 µg/mL) (all from EuroClone SpA Life-Sciences-Division, Milano, Italy). The cells were grown at 37 °C in a humidified atmosphere of 5% CO_2_. When indicated, the cells were treated with H_2_O_2_ (300 µM) for 3 h and with different concentrations (10–1000 µg/mL) of the various extracts of *M. spicata*.

### 3.6. MTT Assay

The cell viability was evaluated after 24 h and 48 h of culturing using the MTT (3-[4,5-dimethyl-thiazol-2-yl-]-2,5-diphenyl tetrazolium bromide) growth assay (Sigma-Aldrich, St. Louis, MO, USA), based on the capability of viable cells to reduce MTT to a colored formazan product. Details about the protocol were reported in our recent study [97].

### 3.7. PGE_2_ ELISA Assay

The HypoE22 rat-hypothalamus cell line was treated with H_2_O_2_ (300 µM) for 3 h and with different concentrations (1–100 µg/mL) of the HAE acetone/water extract of *M. spicata*. At 48 h, the cell supernatants were harvested and the secretion of prostaglandin E_2_ (PGE_2_) in the culture media was evaluated by an ELISA kit (Enzo Life Sciences, Farmingdale, NY, USA), according to the manufacturer’s instructions. The optical density values were obtained by measuring the absorbance at 405 nm using a Multiscan GO microplate spectrophotometer (Thermo Fisher Scientific, Waltham, MA, USA).

### 3.8. Quantitative Determination of Dopamine (DA), Dihydroxyphenilacetic Acid (DOPAC), Levodopa (L-Dopa), Serotonin (5-HT) and 5-Hydroxyindolacetic Acid (5HIIA)

DA, 5-HT, 5HIIA, L-dopa and DOPAC levels were analyzed through a HPLC apparatus consisting of a Jasco (Tokyo, Japan) PU-2080 chromatographic pump and an ESA (Chelmsford, MA, USA) Coulochem III coulometric detector, equipped with a microdialysis cell (ESA-5014b) porous graphite working electrode and a solid-state palladium reference electrode. The detailed description of the chromatographic analysis is fully described in a previous study [98].

### 3.9. Gene Expression Analysis

Gene expression of COX-2 was conducted as previously reported [99]. Briefly, after extraction through the TRI Reagent, total RNA was reverse transcribed using the High Capacity cDNA Reverse Transcription Kit (ThermoFischer Scientific, Waltham, MA, USA). Gene expression was determined by quantitative real-time PCR using TaqMan probes obtained from ThermoFischer Scientific (Waltham, MA, USA). β-actin was used as the house-keeping gene. The analysis of data was carried out with the Sequence Detection System (SDS) software version 2.3 (ThermoFischer Scientific, Waltham, MA, USA). A detailed description of the experimental protocol is reported in a previous paper of ours [99].

### 3.10. In Silico Studies

Putative targets were identified according to the bioinformatics method recently described by Gu et al. [100]. Briefly, proteins targeted by extracts were predicted using the bioinformatics platform SwissTargetPrediction, and the components-targets analysis was conducted through the software Cytoscape (Version 3.8).

### 3.11. Statistical Analysis

The experimental data related to in vitro studies were analyzed through the analysis of variance (ANOVA) followed by Newman–Keuls post hoc test. The GraphPad Prism software (9.0) was employed for statistical analysis. *p* < 0.05 was considered statistically significant.

## 4. Conclusions

This study was intended to explore the phytochemical composition and bio-pharmacological effects of *M. spicata* extracts obtained by different extraction methods and solvents. In general, the acetone/water extracts had the highest content in total phenols and exhibited the strongest antioxidant effects, regardless of the extraction method used. In particular, the HAE extract was able to protect HypoE22 hypothalamic cells from the oxidative stress injury suffered after exposure to hydrogen peroxide. Indeed, the acetone/water extract prevented the hydrogen peroxide-induced dopamine depletion and reduced the production of pro-inflammatory PGE_2_ in vitro. Hence, neuroprotective effects are partly related to the prominent content in the extract of phytochemicals, namely acacetin, eriodictyol, hesperidin, sagerinic acid, naringenin, luteolin, chlorogenic acid, chrysoeriol and apigenin. Based on these findings, the current work provided new scientific bases for anti-inflammatory and neuromodulatory properties of *M. spicata* extract and its potential applications in the pharmaceutical industry. However, further studies are needed to improve our knowledge about the aforementioned effects by the extracts through the use of independent in vitro models, including astrocytes which have been described to support neuron function and response to xenobiotics [101].

## Figures and Tables

**Table 1:** Total phenolic and flavonoid contents in the tested extracts.

Extraction Method	Solvent	Total Phenolic Content	Total Flavonoid Content
		(mg GAE/g)	(mg RE/g)
HAE	Hexane	32.75 ± 0.42 ^c,^*	nd
	Chloroform	27.39 ± 0.95 ^d^	16.44 ± 0.30 ^b^
	Acetone	52.19 ± 0.67 ^b^	14.43 ± 0.70 ^c^
	Acetone/Water	129.68 ± 1.43 ^a^	92.20 ± 0.69 ^a^
UAE	Hexane	11.88 ± 0.29 ^d^	nd
	Chloroform	28.71 ± 1.15 ^c^	18.16 ± 0.66 ^b^
	Acetone	55.95 ± 1.12 ^b^	nd
	Acetone/Water	142.62 ± 3.52 ^a^	84.95 ± 0.76 ^a^
MAC	Hexane	13.15 ± 0.05 ^d^	1.02 ± 0.13 ^b^
	Chloroform	30.99 ± 0.25 ^c^	nd
	Acetone	58.62 ± 1.12 ^b^	nd
	Acetone/Water	87.69 ± 0.38 ^a^	84.42 ± 1.56 ^a^

* Values are reported as mean ± SD of three parallel measurements. GAE: Gallic acid equivalent; RE: Rutin equivalent. HAE: Homogenizer assisted extraction; UAE: Ultrasound assisted extraction; MAC: Maceration. nd: not detected. Different superscript letters within columns indicate significant differences in the tested extracts for the extraction methods (*p* < 0.05).

**Table 2:** Characterization of the compounds found in the analyzed extracts of *Mentha spicata*.

No.	t*_R_*	[M-H]^−^	*m*/*z* (% Base Peak)	Assigned Identification	HAE-Hexane	HAE-Chloroform	HAE-Acetone	HAE-Aceton/Water	UAE-Hexane	UAE-Chloroform	UAE-Acetone	UAE-Aceton/Water	MAC-Hexane	MAC-Chloroform	MAC-Acetone	MAC-Aceton/Water
	(min)	*m*/*z*														
1	1.8	341	MS^2^ [341]: 179 (100), 161 (37), 149 (14), 143 (12), 131 (10), 119 (9), 113 (15), 101 (8)	Disaccharide	+	+	+	+	+	+	+	+	+	+	+	+
2	2.7	191	MS^2^ [191]: 173 (29), 129 (6), 111 (100)	Citric acid *	+	+	+	−	+	+	+	−	+	+	+	+
3	3.8	315	MS^2^ [315]: 153 (100)	Dihydroxybenzoic acid-*O*-hexoside	-	−	+	+	−	−	−	+	−	−	−	+
			MS^3^ [315→153]: 123 (100)													
4	9.0	353	MS^2^ [353]: 191 (25), 179 (60), 173 (100)	Chlorogenic acid *	−	−	+	+	−	−	+	+	−	−	+	+
5	9.8	305	MS^2^ [305]: 225 (100)	Unknown	−	−	−	+	−	−	−	+	−	−	−	+
			MS^3^ [305→225]: 163 (100)													
6	11.2	179	MS^2^ [179]: 135 (100)	Caffeic acid *	-	−	+	−	−	−	−	−	−	−	−	+
7	17.9	595	MS^2^ [595]: 287 (100)	Eriodictyol-*O*-rutinoside	+	+	+	+	+	+	+	+	+	+	+	+
			MS^3^ [595→287]: 151 (100), 135 (8)													
8	19.1	449	MS^2^ [449]: 287 (100), 151 (7)	Eriodictyol-*O*-hexoside	−	−	+	+	−	−	+	+	−	−	+	+
			MS^3^ [449→287]: 151 (100), 135 (12)													
9	19.8	497	MS^2^ [497]: 451 (100)	Unknown	+	+	+	−	+	+	+	−	+	+	+	−
			MS^3^ [497→451]: 225 (100)													
10	20.1	609	MS^2^ [609]: 301 (100)	Rutin *	−	−	+	+	−	−	−	+	−	−	−	+
			MS^3^ [609→301]: 179 (100), 151 (85)													
11	20.4	593	MS^2^ [593]: 285 (100)	Luteolin-*O*-rutinoside	+	+	+	+	+	−	+	+	+	+	+	+
			MS^3^ [593→285]: 285 (100), 243(17), 241 (38)													
12	21.5	447	MS^2^ [447]: 285 (100)	Luteolin-*O*-hexoside	+	+	+	+	−	−	+	+	+	+	+	+
			MS^3^ [447→285]: 285 (100), 243 (24), 241 (18)													
13	21.5	461	MS^2^ [461]: 285 (100)	Luteolin-*O*-glucuronide	+	−	+	+	−	−	−	+	+	+	−	+
			MS^3^ [461→285]: 285 (100), 241 (15)													
14	22.1	579	MS^2^ [579]: 271 (100)	Naringenin-*O*-rutinoside	+	−	+	+	−	−	+	+	−	−	+	+
			MS^3^ [579→271]: 177 (25), 151 (100)													
15	24.0	577	MS^2^ [577]: 269 (100)	Apigenin-*O*-rutinoside	+	−	+	+	−	+	+	+	+	+	+	+
			MS^3^ [577→269]: 225 (100)													
16	24.3	609	MS^2^ [609]: 301 (100)	Hesperidin *	+	+	+	+	−	+	+	+	−	+	+	+
			MS^3^ [609→301]: 286 (34), 283 (57), 241 (100), 227 (58), 125 (58)													
17	25.1	717	MS^2^ [717]: 537 (38), 519 (100)	Salvianolic acid B/E/L	−	−	−	+	−	−	−	+	−	−	−	+
			MS^3^ [717→519]: 339 (100), 321 (14), 295 (7), 277 (7)													
18	25.4	431	MS^2^ [431]: 269 (100)MS^3^ [431→269]: 225 (100), 151 (49)	Apigenin-*O*-hexoside	+	−	−	+	−	+	+	+	+	+	+	+
19	25.5	607	MS^2^ [607]: 299 (100), 284 (43)	Chrysoeriol-*O*-rutinoside	+	−	+	+	−	−	+	+	−	−	+	+
			MS^3^ [607→299]: 284 (100)													
20	26.1	719	MS^2^ [719]: 359 (100)	Sagerinic acid	+	−	+	+	−	−	+	+	−	−	+	+
			MS^3^ [719→359]: 197 (18), 179 (25), 161 (100), 135 (2)													
21	29.6	537	MS^2^ [537]: 493 (100), 359 (25)	Salvianolic acid I	−	−	−	+	−	+	+	+	−	+	+	+
			MS^3^ [537→493]: 359 (100), 179 (12), 161 (10)													
22	33.5	591	MS^2^ [591]: 283 (100), 268 (16)	Acacetin-*O*-rutinoside	−	−	+	+	−	−	+	+	−	−	+	+
23	36.0	285	MS^2^ [285]: 285 (100), 243 (28), 241 (8)	Luteolin *	−	−	+	+	−	−	+	−	−	−	+	+
24	37.3	299	MS^2^ [299]: 284 (100)	Chrysoeriol	−	+	+	−	−	+	+	−	−	+	+	+
25	38.2	551	MS^2^ [551]: 519 (100), 359 (60)	Monomethyl lithospermate	−	−	+	+	−	−	+	+	−	−	+	+
			MS^3^ [551→519]: 339 (100), 179 (28), 161 (23)													
26	39.1	327	MS^2^ [327]: 291 (31), 229 (100), 221 (16), 211 (46), 171 (52)	Oxo-dihydroxy-octadecenoic acid	−	+	+	+	−	+	+	+	+	+	+	−
27	40.5	329	MS^2^ [329]: 311 (23), 293 (35), 229 (100), 211 (58)	Trihydroxy-octadecenoic acid	−	+	+	+	−	+	+	+	+	+	+	−

* Identified by comparison with analytical standards. HAE: Homogenizer assisted extraction; UAE: Ultrasound assisted extraction; MAC: Maceration. −: not detected; +: detected.

**Table 3:** Quantification of the main compounds detected in *Mentha spicata* (mg g^−1^ DE).

N°	Assigned Identification	HAE-Acet	HAE-Acet: H_2_O	UAE-Acet	UAE-Acet: H_2_O	MAC-Acet	MAC-Acet: H_2_O
*Phenolic acids*							
4	Chlorogenic acid	0.19 ± 0.01	3.4 ± 0.2	0.15 ± 0.01	2.9 ± 0.2	0.14 ± 0.01	2.4 ± 0.2
20	Sagerinic acid	4.2 ± 0.3	30 ± 2	2.4 ± 0.2	29 ± 2	8.7 ± 0.5	17 ± 1
Total		4.4 ± 0.3	33 ± 2	2.6 ± 0.2	32 ± 2	8.8 ± 0.5	19 ± 1
*Flavonoids*							
7	Eriodictyol-*O*-rutinoside	26 ± 2	74 ± 5	14.8 ± 0.9	75 ± 4	62 ± 3	69 ± 4
8	Eriodictyol-*O*-hexoside	0.35 ± 0.03	12.6 ± 0.9	0.42 ± 0.03	15 ± 1	5.9 ± 0.4	15 ± 1
11	Luteolin-*O*-rutinoside	1.3 ± 0.1	7.4 ± 0.5	0.47 ± 0.03	7.5 ± 0.5	2.4 ± 0.2	6.8 ± 0.5
12+13	Luteolin glycosides	2.1 ± 0.1	25 ± 2	0.65 ± 0.04	25 ± 2	3.1 ± 0.2	26 ± 1
14	Naringenin-*O*-rutinoside	6.4 ± 0.4	6.8 ± 0.5	0.22 ± 0.02	9.4 ± 0.6	14 ± 1	8.2 ± 0.5
15	Apigenin-*O*-rutinoside	1.5 ± 0.1	4.3 ± 0.3	0.84 ± 0.05	4.2 ± 0.3	3.0 ± 0.2	4.6 ± 0.3
16	Hesperidin	1.7 ± 0.1	9.8 ± 0.7	0.21 ± 0.01	11.8 ± 0.7	5.4 ± 0.3	10.1 ± 0.7
18+19	Apigenin+ chrysoeriol glycosides	1.2 ± 0.1	3.1 ± 0.2	0.57 ± 0.04	3.3 ± 0.2	2.2 ± 0.1	3.4 ± 0.2
22	Acacetin-*O*-rutinoside	0.54 ± 0.04	0.97 ± 0.07	0.36 ± 0.02	0.91 ± 0.06	0.91 ± 0.06	0.89 ± 0.06
23	Luteolin	0.72 ± 0.05	0.88 ± 0.06	0.36 ± 0.02	0.82 ± 0.05	0.71 ± 0.05	1.2 ± 0.1
24	Chrysoeriol	0.68 ± 0.05	0.33 ± 0.02	0.45 ± 0.03	0.30 ± 0.02	0.61 ± 0.04	0.28 ± 0.02
Total		43 ± 2	145 ± 6	19 ± 1	153 ± 5	100 ± 3	145 ± 4
TIPC		47 ± 2	178 ± 6	22 ± 1	185 ± 5	109 ± 3	164 ± 4

TIPC = Total Individual Phenolic Content (sum of all quantified compounds by HPLC-UV). HAE: Homogenizer assisted extraction; UAE: Ultrasound assisted extraction; MAC: Maceration.

**Table 4:** Antioxidant properties of the tested extracts *.

Extraction Methods	Solvent	DPPH (mg TE/g)	ABTS (mg TE/g)	CUPRAC (mg TE/g)	FRAP (mg TE/g)	PBD (mmol TE/g)	Metal Chelating (mg EDTAE/g)
HAE	Hexane	33.15 ± 0.69 ^c^	38.16 ± 1.13 ^c^	82.87 ± 1.67 ^c^	41.71 ± 0.57 ^c^	3.23 ± 0.15 ^a^	24.09 ± 3.37 ^a^
	Chloroform	11.84 ± 0.70 ^d^	29.63 ± 1.12 ^c^	94.12 ± 0.76 ^c^	37.34 ± 1.25 ^c^	2.14 ± 0.11 ^c^	3.30 ± 0.29 ^b^
	Acetone	84.88 ± 0.62 ^b^	84.43 ± 1.62 ^b^	200.27 ± 3.38 ^b^	100.15 ± 1.45 ^b^	2.67 ± 0.14 ^b^	21.80 ± 1.00 ^a^
	Acetone/Water	419.18 ± 1.52 ^a^	348.78 ± 6.48 ^a^	678.48 ± 17.75 ^a^	458.51 ± 8.09 ^a^	2.91 ± 0.02 ^b^	7.98 ± 1.19 ^b^
UAE	Hexane	7.33 ± 0.25 ^d^	9.11 ± 0.83 ^d^	37.72 ± 0.81 ^d^	22.69 ± 0.50 ^d^	0.76 ± 0.07 ^d^	na
	Chloroform	17.46 ± 0.67 ^c^	32.71 ± 2.30 ^c^	102.70 ± 0.53 ^c^	42.45 ± 0.63 ^c^	1.97 ± 0.05 ^c^	11.32 ± 0.96 ^b^
	Acetone	90.29 ± 0.12 ^b^	100.54 ± 2.11 ^b^	248.13 ± 4.83 ^b^	123.43 ± 2.55 ^b^	2.64 ± 0.11 ^b^	21.54 ± 2.58 ^a^
	Acetone/Water	427.29 ± 2.35 ^a^	392.44 ± 14.91 ^a^	782.24 ± 9.99 ^a^	492.27 ± 14.05 ^a^	3.48 ± 0.36 ^a^	8.86 ± 3.84 ^b^
MAC	Hexane	7.53 ± 0.40 ^d^	8.38 ± 1.34 ^d^	42.32 ± 1.38 ^d^	24.16 ± 0.84 ^d^	1.03 ± 0.03 ^c^	15.49 ± 1.04 ^b^
	Chloroform	12.99 ± 0.76 ^c^	32.03 ± 1.92 ^c^	101.13 ± 1.11 ^c^	41.59 ± 0.83 ^c^	2.38 ± 0.17 ^a^	na
	Acetone	89.71 ± 0.22 ^b^	99.70 ± 1.41 ^b^	239.91 ± 2.05 ^b^	119.14 ± 3.25 ^b^	2.54 ± 0.19 ^a^	20.18 ± 0.92 ^a^
	Acetone/Water	266.60 ± 3.09 ^a^	228.82 ± 8.90 ^a^	456.73 ± 3.56 ^a^	302.10 ± 5.33 ^a^	1.90 ± 0.04 ^b^	9.53 ± 1.80 ^c^

* Values are reported as mean ± SD of three parallel measurements. TE: Trolox equivalent. EDTAE: EDTA equivalent. HAE: Homogenizer assisted extraction; UAE: Ultrasound assisted extraction; MAC: Maceration. na: not active. Different superscript letters within columns indicate significant differences in the tested extracts for the extraction method (*p* < 0.05).

**Table 5:** Enzyme inhibitory effects of the tested extracts ^*^.

Extraction Methods	Solvent	AChE (mg GALAE/g)	BChE (mg GALAE/g)	Tyrosinase (mg KAE/g)	Amylase (mmol ACAE/g)	Glucosidase (mmol ACAE/g)
HAE	Hexane	3.77 ± 0.27 ^ab^	8.70 ± 1.29 ^a^	95.99 ± 4.83 ^ab^	0.83 ± 0.01 ^a^	0.62 ± 0.06 ^d^
	Chloroform	4.18 ± 0.39 ^a^	6.50 ± 0.24 ^b^	91.82 ± 3.81 ^ab^	0.71 ± 0.03 ^b^	1.40 ± 0.03 ^b^
	Acetone	4.30 ± 0.36 ^a^	5.61 ± 0.77 ^b^	87.41 ± 7.58 ^b^	0.86 ± 0.03 ^a^	0.90 ± 0.03 ^c^
	Acetone/Water	3.11 ± 0.28 ^b^	2.12 ± 0.13 ^c^	101.08 ± 1.78 ^a^	0.61 ± 0.04 ^c^	1.66 ± 0.01 ^a^
UAE	Hexane	na	4.36 ± 0.80 ^b^	85.33 ± 9.38 ^b^	0.57 ± 0.01 ^b^	1.27 ± 0.03 ^bc^
	Chloroform	4.84 ± 0.20 ^a^	7.62 ± 0.97 ^a^	98.81 ± 3.10 ^ab^	0.80 ± 0.01 ^a^	1.39 ± 0.03 ^ab^
	Acetone	4.30 ± 0.20 ^b^	5.24 ± 0.18 ^b^	107.67 ± 4.34 ^a^	0.83 ± 0.01 ^a^	1.20 ± 0.01 ^c^
	Acetone/Water	4.21 ± 0.03 ^b^	1.33 ± 0.11 ^c^	100.75 ± 1.06 ^a^	0.57 ± 0.02 ^b^	1.52 ± 0.12 ^a^
MAC	Hexane	na	7.49 ± 0.44 ^a^	96.03 ± 5.33 ^bc^	0.54 ± 0.01 ^b^	1.29 ± 0.03 ^c^
	Chloroform	4.82 ± 0.36 ^a^	6.69 ± 0.61 ^ab^	95.03 ± 1.93 ^c^	0.75 ± 0.01 ^a^	1.49 ± 0.02 ^b^
	Acetone	2.85 ± 0.16 ^b^	5.46 ± 0.76 ^b^	103.61 ± 2.81 ^ab^	0.74 ± 0.01 ^a^	1.45 ± 0.03 ^b^
	Acetone/Water	3.37 ± 0.20 ^b^	na	108.38 ± 1.52 ^a^	0.52 ± 0.01 ^b^	1.62 ± 0.01 ^a^

* Values are reported as mean ± SD of three parallel measurements. GALAE: Galatamin equivalent; KAE: Kojic acid equivalent; ACAE: Acarbose equivalent; HAE: Homogenizer assisted extraction; UAE: Ultrasound assisted extraction; MAC: Maceration. na: not active. Different superscript letters within columns indicate significant differences in the tested extracts for the extraction methods (*p* < 0.05).

## Data Availability

Data is contained within the article and supplementary material.

## Supplementary Materials

The following are available online at https://www.mdpi.com/article/10.3390/plants11020233/s1, Table S1: Comparison of extracts by homogenizer assisted extraction, Table S2: Comparison of extracts by ultrasound assisted extraction, Table S3: Comparison of extracts by maceration extraction, Table S4: Comparison of acetone/water extracts by the tested extraction methods.
