# Supplementary material for: Novel Perceptions on Chemical Profile and Biopharmaceutical Properties of Mentha spicata Extracts: Adding Missing Pieces to the Scientific Puzzle
Source: Plants (Basel). 2022 Jan 17;11(2):233. doi: 10.3390/plants11020233 (PMC8779166; doi:10.3390/plants11020233)
Supplement: Supplementary file 1 [file plants-11-00233-s001.zip › plants-1555144-supplementary.pdf]

Article

Novel Perceptions on Chemical Profile and Biopharmaceutical Properties of *Mentha spicata* Extracts: Adding Missing Pieces to the Scientific Puzzle

Gokhan Zengin[1], Gunes Ak[1], Ramazan Ceylan[1], Sengul Uysal[2,3], Eulogio Llorent-Martínez[4], Simonetta Cristina Di Simone[5], Monica Rapino[6], Alessandra Acquaviva[5], Maria Loreta Libero[5], Annalisa Chiavaroli[5], Lucia Recinella[5], Sheila Leone[5], Luigi Brunetti[5], Amelia Cataldi[5], Giustino Orlando[5], Luigi Menghini[5], Claudio Ferrante[5]*, Marwa Balaha[5,7] and Viviana di Giacomo[5]

[1] Physiology and Biochemistry Research Laboratory, Department of Biology, Science Faculty, Selcuk University, Campus, Konya, Turkey. gokhanzengin@selcuk.edu.tr (G.Z.); akguneselcuk@gmail.com (G.A); biyoram7@gmail.com (R.C)
[2] Erciyes University Halil Bayraktar Health Services Vocational College, Kayseri, Turkey. senguluysal@erciyes.edu.tr (S.U)
[3] Drug Application and Research Center, Erciyes University, Kayseri, Turkey
[4] Department of Physical and Analytical Chemistry, Campus Las Lagunillas S/N, University of Jaén,E-23071 Jaén, Spain; ellorent@ujaen.es (E.L.M.);
[5] Department of Pharmacy, Botanic Garden "Giardino dei Semplici", "Gabriele d'Annunzio" University, via dei Vestini 31, 66100 Chieti, Italy; simonetta.disimone@unich.it (S.C.D.S.); alessandra.acquaviva@unich.it (A.A); maria.libero@unich.it (M.L.L.); annalisa.chiavaroli@unich.it (A.C.); lucia.recinella@unich.it (L.R.); sheila.leone@unich.it (S.L.); luigi.brunetti@unich.it (L.B.); amelia.cataldi@unich.it (A.C.); giustino.orlando@unich.it (G.O.); claudio.ferrante@unich.it (C.F.), marwa.balaha@unich.it (M. B.), viviana.digiacomo@unich.it (V.d.G.)
[6] Genetic Molecular Institute of CNR, Unit of Chieti, "G. d' Annunzio" University, Via dei Vestini 31, 66100 Chieti, Italy; m.rapino@unich.it (M.R.);
[7] Department of Pharmaceutical Chemistry, Faculty of Pharmacy, Kafrelsheikh University, Kafr El Sheikh 33516, Egypt.
* Correspondence: claudio.ferrante@unich.it (C.F.)

Citation: Zengin, G.; Ak, G.; Ceylan, R.; Uysal, S.; Llorent-Martínez, E.; Di Simone, S.C.; Rapino, M.; Acquaviva, A.; Libero, M.L.; Chiavaroli, A.; et al. Novel Perceptions on Chemical Profile and Biopharmaceutical Properties of Mentha spicata Extracts: Adding Missing Pieces to the Scientific Puzzle. *Plants* **2022**, *11*, 233. https://doi.org/10.3390/plants11020233

Academic Editor: Maria José U. Ferreira

Received: 29 December 2021
Accepted: 13 January 2022
Published: 17 January 2022

Publisher's Note: MDPI stays neutral with regard to jurisdictional claims in published maps and institutional affiliations.

Copyright: © 2022 by the authors. Licensee MDPI, Basel, Switzerland. This article is an open access article distributed under the terms and conditions of the Creative Commons Attribution (CC BY) license (http://creativecommons.org/licenses/by/4.0/).

Table S1. Comparison of extracts by homogenizer assisted extraction

Extracts	Total compounds	Compounds
HAE-Acetone<br>HAE-Acetone/Water<br>HAE-Chloroform<br>HAE-Hexan	5	Disaccharide, Luteolin-O-hexoside, Luteolin-O-rutinoside, Eriodictyol-O-rutinoside, Hesperidin*
HAE-Acetone<br>HAE-Chloroform<br>HAE-Hexan	2	Citric acid*, Unknown-2
HAE-Acetone<br>HAE-Acetone/Water<br>HAE-Hexan	5	Chrysoeriol-O-rutinoside, Luteolin-O-glucuronide, Sagerinic acid, Apigenin-O-rutinoside, Naringenin-O-rutinoside
HAE-Acetone<br>HAE-Acetone/Water<br>HAE-Chloroform	2	Trihydroxy-octadecenoic acid, Oxo-dihydroxy-octadecenoic acid
HAE-Acetone/Water<br>HAE-Hexan	1	Apigenin-O-hexoside
HAE-Acetone<br>HAE-Chloroform	1	Chrysoeriol
HAE-Acetone<br>HAE-Acetone/Water	7	Rutin*, Acacetin-O-rutinoside, Eriodictyol-O-hexoside, Chlorogenic acid*, Luteolin*, Monomethyl lithospermate, Dihydroxybenzoic acid-O-hexoside
HAE-Acetone	1	Caffeic acid*
HAE-Acetone/Water	3	Salvianolic acid I, Unknown-1, Salvianolic acid B/E/L

Table S2. Comparison of extracts by ultrasound assisted extraction

Extracts	Total compounds	Compounds
UAE-Acetone<br>UAE-Acetone/Water<br>UAE-Chloroform<br>UAE-Hexan	2	Disaccharide, Eriodictyol-O-rutinoside
UAE-Acetone<br>UAE-Chloroform<br>UAE-Hexan	2	Citric acid*, Unknown-2
UAE-Acetone<br>UAE-Acetone/Water<br>UAE-Hexan	1	Luteolin-O-rutinoside
UAE-Acetone<br>UAE-Acetone/Water<br>UAE-Chloroform	6	Salvianolic acid I, Trihydroxy-octadecenoic acid, Apigenin-O-rutinoside, Hesperidin*, Apigenin-O-hexoside, Oxo-dihydroxy-octadecenoic acid
UAE-Acetone<br>UAE-Chloroform	1	Chrysoeriol
UAE-Acetone<br>UAE-Acetone/Water	8	Chrysoeriol-O-rutinoside, Acacetin-O-rutinoside, Eriodictyol-O-hexoside, Luteolin-O-hexoside, Chlorogenic acid*, Sagerinic acid Monomethyl lithospermate, Naringenin-O-rutinoside
UAE-Acetone	1	Luteolin*
UAE-Acetone/Water	5	Rutin*, Luteolin-O-glucuronide, Unknown-1, Salvianolic acid B/E/L , Dihydroxybenzoic acid-O-hexoside

Table S3. Comparison of extracts by maceration extraction

Extracts	Total compounds	Compounds
MAC-Acetone<br>MAC-Acetone/Water<br>MAC-Chloroform<br>MAC-Hexan	7	Disaccharide, Luteolin-O-hexoside, Citric acid*, Luteolin-O-rutinoside, Apigenin-O-rutinoside, Eriodictyol-O-rutinoside, Apigenin-O-hexoside
MAC-Acetone<br>MAC-Chloroform<br>MAC-Hexan	3	Trihydroxy-octadecenoic acid, Unknown-2, Oxo-dihydroxy-octadecenoic acid
MAC-Acetone/Water<br>MAC-Chloroform<br>MAC-Hexan	1	Luteolin-O-glucuronide
MAC-Acetone<br>MAC-Acetone/Water<br>MAC-Chloroform	3	Chrysoeriol, Salvianolic acid, I Hesperidin*
MAC-Acetone<br>MAC-Acetone/Water	8	Chrysoeriol-O-rutinoside, Acacetin-O-rutinoside, Eriodictyol-O-hexoside, Chlorogenic acid*, Sagerinic acid, Luteolin*, Monomethyl lithospermate, Naringenin-O-rutinoside
MAC-Acetone/Water	5	Rutin*, Unknown-1, Salvianolic acid B/E/L, Caffeic acid*, Dihydroxybenzoic acid-O-hexoside

Table S4. Comparison of acetone/water extracts by the tested extraction methods

Methods	Total compounds	Compounds
HAE<br>MAC<br>UAE	20	Chrysoeriol-O-rutinoside, Rutin*, Disaccharide, Acacetin-O-rutinoside, Eriodictyol-O-hexoside, Luteolin-O-hexoside, Salvianolic acid I, Luteolin-O-glucuronide, Chlorogenic acid*, Sagerinic acid, Unknown-1, Luteolin-O-rutinoside, Apigenin-O-rutinoside, Eriodictyol-O-rutinoside, Hesperidin*, Monomethyl lithospermate, Salvianolic acid B/E/L, Apigenin-O-hexoside, Naringenin-O-rutinoside, Dihydroxybenzoic acid-O-hexoside
HAE<br>UAE	2	Trihydroxy-octadecenoic acid, Oxo-dihydroxy-octadecenoic acid
HAE<br>MAC	1	Luteolin*
MAC	3	Chrysoeriol, Citric acid*, Caffeic acid*
